# Microsurgical ligation for incompletely coiled or recurrent intracranial aneurysms: a 17-year single-center experience

**DOI:** 10.1186/s41016-019-0153-z

**Published:** 2019-03-07

**Authors:** Jun Wu, Xianzeng Tong, Qingyuan Liu, Yong Cao, Yuanli Zhao, Shuo Wang

**Affiliations:** 1 Department of Neurosurgery, Beijing Tiantan Hospital, Capital Medical University, Beijing, 100050 China; 2 China National Clinical Research Center for Neurological Diseases, Beijing, China; 3 Center of Stroke, Beijing Institute for Brain Disorders, Beijing, China; 4 Beijing Key Laboratory of Translational Medicine for Cerebrovascular Diseases, Beijing, People’s Republic of China; 5 Department of Neurosurgery, Xuanwu Hospital, Capital Medical University, Beijing, China

**Keywords:** Coiled aneurysms, Recurrent aneurysms, Endovascular coiling, Microsurgical clipping

## Abstract

**Background:**

In this retrospective single-center study, we presented our experience in the microsurgical management of incompletely coiled or recurrent aneurysms after initial endovascular coiling.

**Methods:**

During a 17-year period, 48 patients underwent microsurgical clipping of incompletely coiled or recurrent aneurysms after coiling (Gurian group B). The clinical data, surgical technique, and postoperative outcome were recorded and analyzed.

**Results:**

Before coiling, 42 patients (87.5%) experienced aneurysm rupture. Most of the aneurysms (46/48, 96%) were located in the anterior circulation. After coiling, 6 patients had incompletely coiled aneurysms and 42 patients had recurrent aneurysms, with a mean time of 20.2 months from coiling to recurrence. Coil extrusion occurred in none of the incompletely coiled aneurysms and 71% (30/42) of the recurrent aneurysms. Clipping techniques are direct microsurgical clipping without coil removal in 16 patients, partial coil removal in 14 patients, and total coil removal in 18 patients. Postoperative and follow-up angiography revealed complete occlusion of the aneurysms in all patients. No patient died during postoperative follow-up period (mean, 78.9 months; range, 10–190 months). Good outcomes (GOS of 4 or 5) were achieved in 87.5% (42/48) of the patients at the final follow-up.

**Conclusions:**

Microsurgical clipping is effective for incompletely coiled or recurrent aneurysms after initial coiling. For recurrent aneurysms that have coils in the neck, have no adequate neck for clipping, or cause mass effects on surrounding structures, partial or total removal of coiled mass can facilitate surgical clipping and lead to successful obliteration of the aneurysms.

## Background

Incompletely coiled or recurrent aneurysms after embolization have been reported since the use of coil in aneurysm treatment. The reported rate of aneurysm recurrence after initial endovascular therapy was between 3.6 to 40% [5, 8, 21, 32, 35]. The International Subarachnoid Aneurysm Trial (ISAT) reported a combined rate of 34% for aneurysm incomplete obliteration and recurrence after initial endovascular coiling [21]. Subsequent follow-up data in ISAT showed that 191 (17.4%) out of 1096 patients required retreatment for incomplete occlusion or recurrence and 103 patients (54%) required neurosurgical management [5]. In the Cerebral Aneurysm Rerupture After Treatment (CARAT) study, the annual retreatment rates of coiled aneurysms were 13.3%, 4.5%, and 1.1% respectively during the first, second, and subsequent years [17]. Incompletely obliterated or recurrent aneurysms were more likely to rupture or cause mass effect symptoms than the completely obliterated aneurysms [6–9, 17, 18, 27].

Microsurgical ligation is a treatment strategy for incompletely coiled or recurrent intracranial aneurysm. Surgical management of these aneurysms is challenging. In some cases, the surgical clipping might be the optimal choice. In this study, we retrospectively analyzed our experience of the surgical clipping for incompletely coiled or recurrent aneurysms in our center.

## Methods

### Patients inclusion

The patients that underwent microsurgical ligation of incompletely coiled or recurrent aneurysms after endovascular coiling were searched from the Beijing Tiantan Hospital Cerebral Aneurysm Surgical Treatment database from January 2000 to January 2017. Only the patients with incomplete obliteration or recurrence after coiling (Gurian group B) who received subsequent open surgery were included in this study. Patients were excluded with a failed endovascular treatment with no embolic coils used (Gurian group A) or surgery for the complications related to endovascular therapy (Gurian group C) [14].

### Recurrence and therapeutic decision

After initial endovascular coiling, the patients were followed-up to evaluate the efficacy of initial coiling. The follow-up imaging including magnetic resonance angiography (MRA) and digital subtraction angiography (DSA). Recurrence latency was defined as the time interval between the initial endovascular coiling and the time when the follow-up image clinically showed recurrence.

For incompletely obliterated or recurrent aneurysms after coiling, the treatment strategy was made by a multidisciplinary team including vascular neurosurgeons and interventional neuroradiologists.

### Surgical technique

All surgical procedures were performed by either of two experienced vascular neurosurgeons in our center. Coil removal was indicated when the coils protruded into aneurysm neck or parental arteries that hamper satisfactory surgical clipping. Coil removal was also indicated to avoid stenosis of the parental arteries when there was no adequate neck for clipping. For the recurrent aneurysms that caused mass effects, the coils need to be partially or totally removed. Considering that large or giant aneurysms (size ≥ 10 mm) may cause potential mass effects, we also performed partial or total removal of the coils. For the aneurysms that were small-sized but had adequate neck for clipping (without coil migration into the neck), direct clipping without coil removal was performed. Direct clipping was also used for the aneurysms that were difficult to dissect but had adequate neck for clipping (without coil migration into the neck). None of our patients underwent bypass surgery, parent artery occlusion, or wrapping. Microvascular Doppler ultrasonography or indocyanine green video angiography was used during surgery to verify the exclusion of aneurysms and preservation of branches.

### Follow-up and outcome evaluation

All patients in this series underwent postoperative angiography 3 to 7 days after surgical clipping to document complete obliteration of the aneurysms. Follow-up DSA or MRA was performed at 6 months after surgery and annual. Neurological assessments were performed preoperatively, postoperatively, and during the follow-up period. The Glasgow Outcome Scale (GOS) score was used to define clinical outcomes. Good outcomes were defined as a GOS of 4 or 5 at the final follow-up evaluation and poor outcomes were defined as a final GOS of less than 4. The changes in GOS score from the preoperative to final follow-up evaluation were also documented.

### Data analysis

Statistical analyses were performed with the Statistical Package for the Social Sciences software (version 20.0; SPSS, Inc., Chicago, Illinois). Patient demographics (age at surgery, sex, presentation), aneurysm characteristics (side, size, location, initial result after coiling, recurrent latency, and DSA aspect of the recurrence), intraoperative and postoperative results (surgical technique and follow-up data), and neurological status (GOS) were entered into a spreadsheet. All data were summarized using descriptive statistics for continuous variables (mean ± standard deviation, minimum and maximum) and categorical variables (count and percentage).

## Results

### Initial patient presentation and aneurysm characteristics

**Fig. 1:**
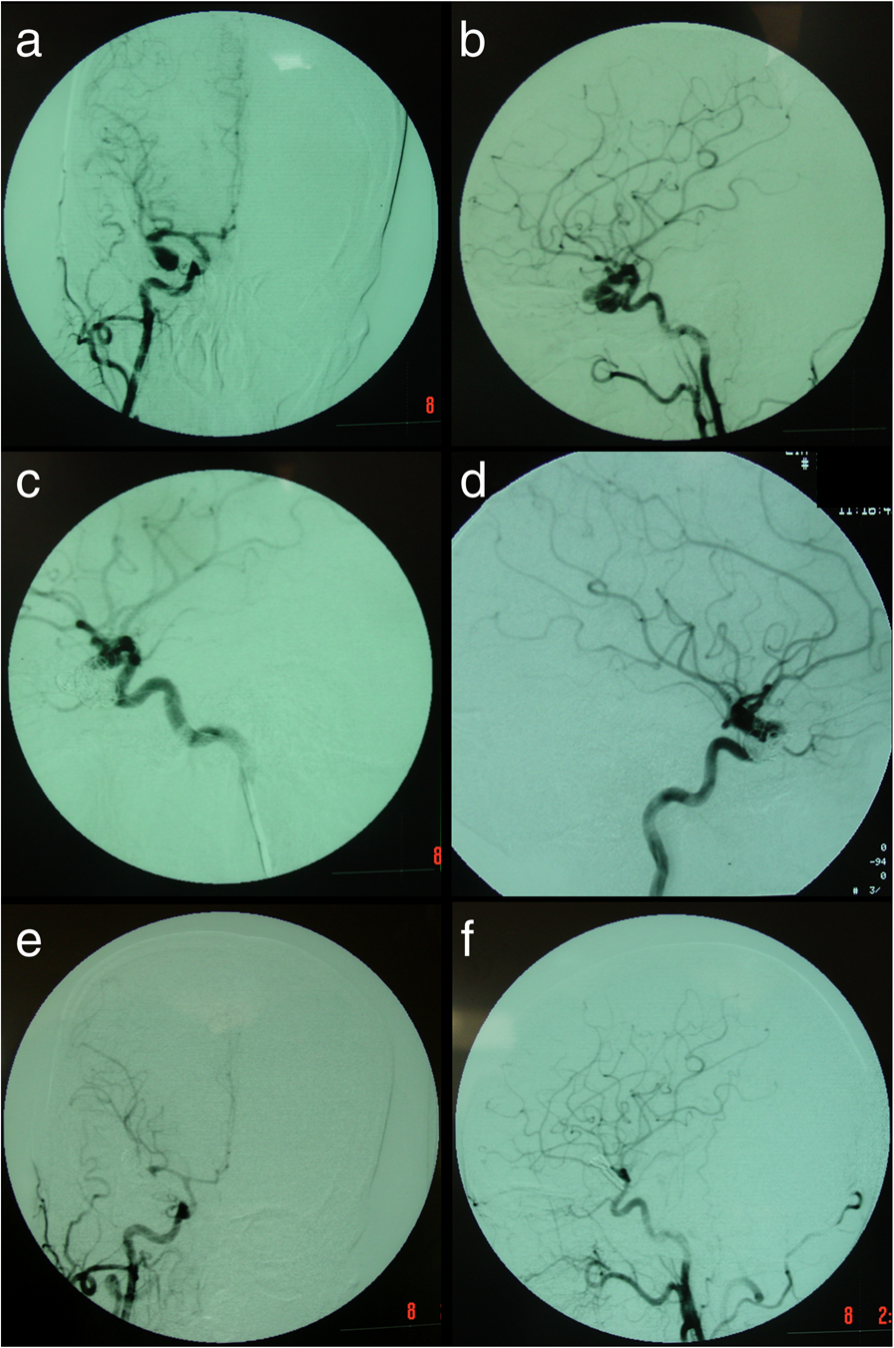
Patient 1. A 47-year-old woman presented with a ruptured right MCA aneurysm (**a**, **b**). She was initially treated with endovascular coiling, and the aneurysm was completely obliterated (**c**). The follow-up DSA revealed an aneurysm recurrence 36 months after initial coil embolization (**d**). She was treated with microsurgical clipping and total coil removal. Postoperative DSA showed complete obliteration of the recurrent aneurysm (**e**, **f**)

**Fig. 2:**
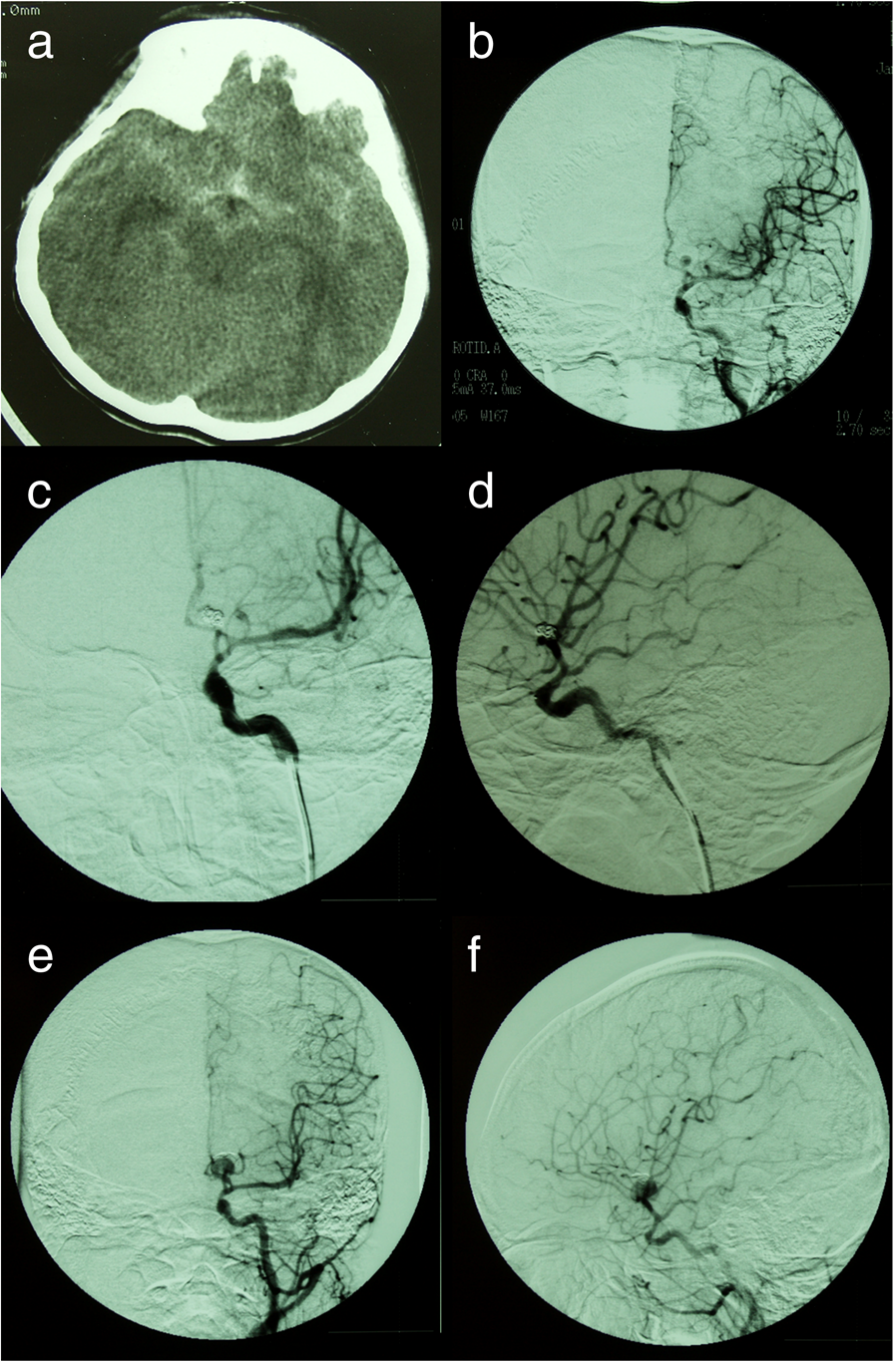
Patient 2. A 12-year-old boy presented with a ruptured left ACA-A1 aneurysm (**a**, **b**). Complete obliteration was achieved after initial coiling (**c**, **d**). The follow-up DSA showed an aneurysm recurrence 3 months after initial coiling (**e**, **f**)

**Fig. 3:**
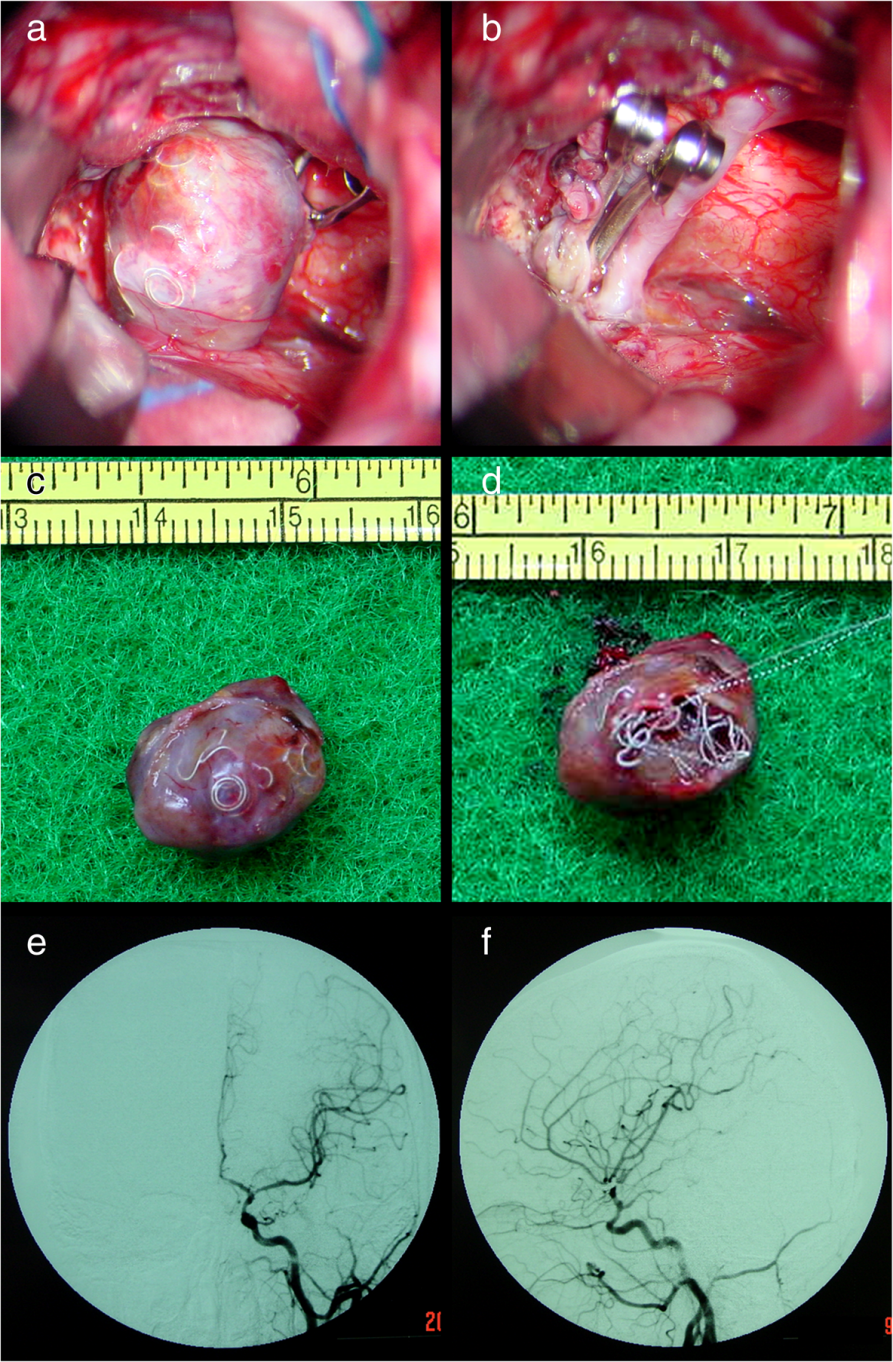
Patient 2. Coil compaction was observed during surgery (**a**). The aneurysm was successfully clipped and the coiled mass was totally removed (**b**-**d**). Postoperative DSA revealed complete obliteration of the recurrent aneurysm (**e**, **f**)

**Fig. 4:**
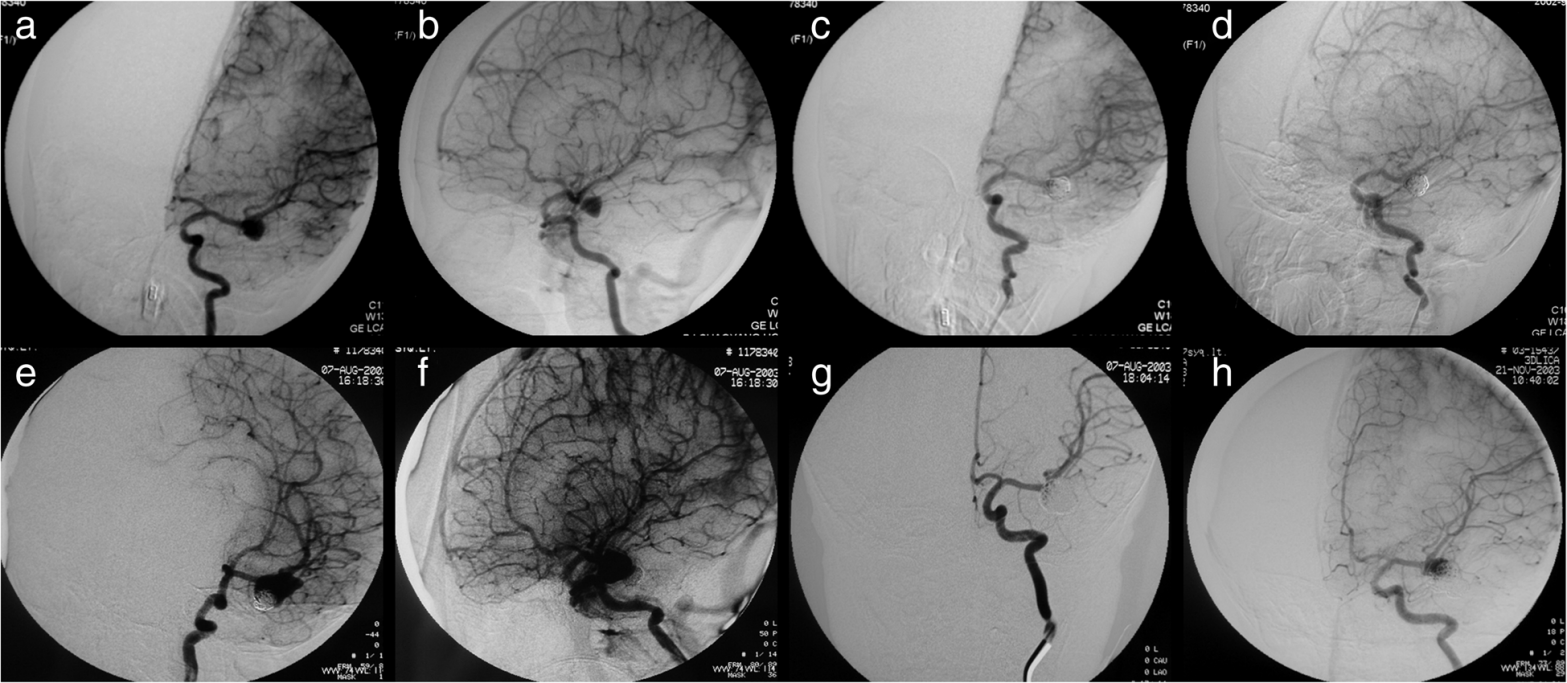
Patient 5. A 3-year-old boy presented with an unruptured left MCA aneurysm (**a**, **b**). The aneurysm was completely obliterated after initial coiling (**c**, **d**). The follow-up DSA showed an aneurysm recurrence 11 months after initial coiling (**e**, **f**). At the same time, the recurrent aneurysm was completely obliterated with recoiling (**g**). Three and a half months after recoiling, follow-up DSA showed a second recurrence of the coiled aneurysm (**h**)

**Fig. 5:**
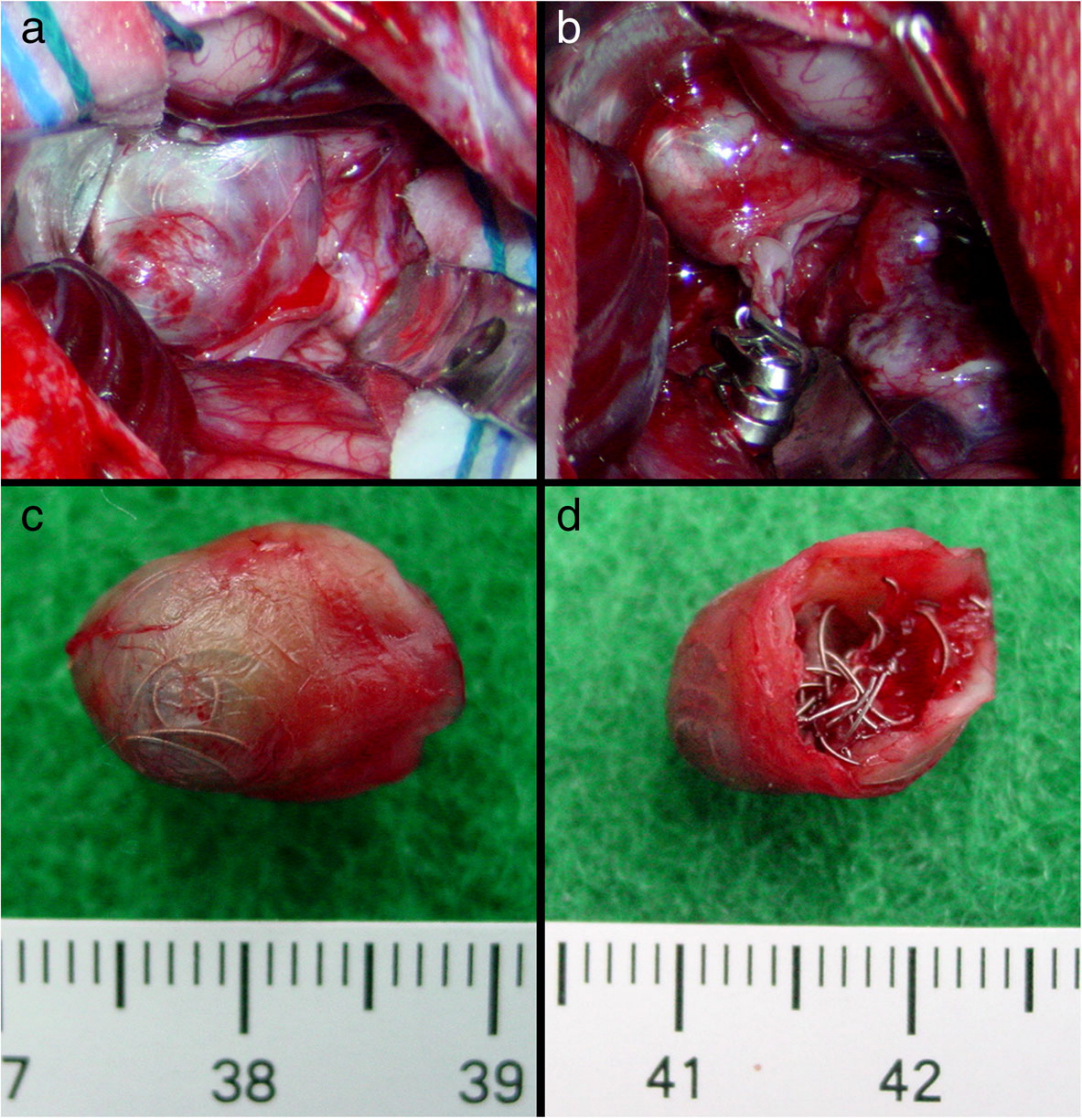
Patient 5. Coil compaction was observed during surgery (**a**). The aneurysm was successfully occluded and the coiled mass was totally removed (**b**-**d**)

**Fig. 6:**
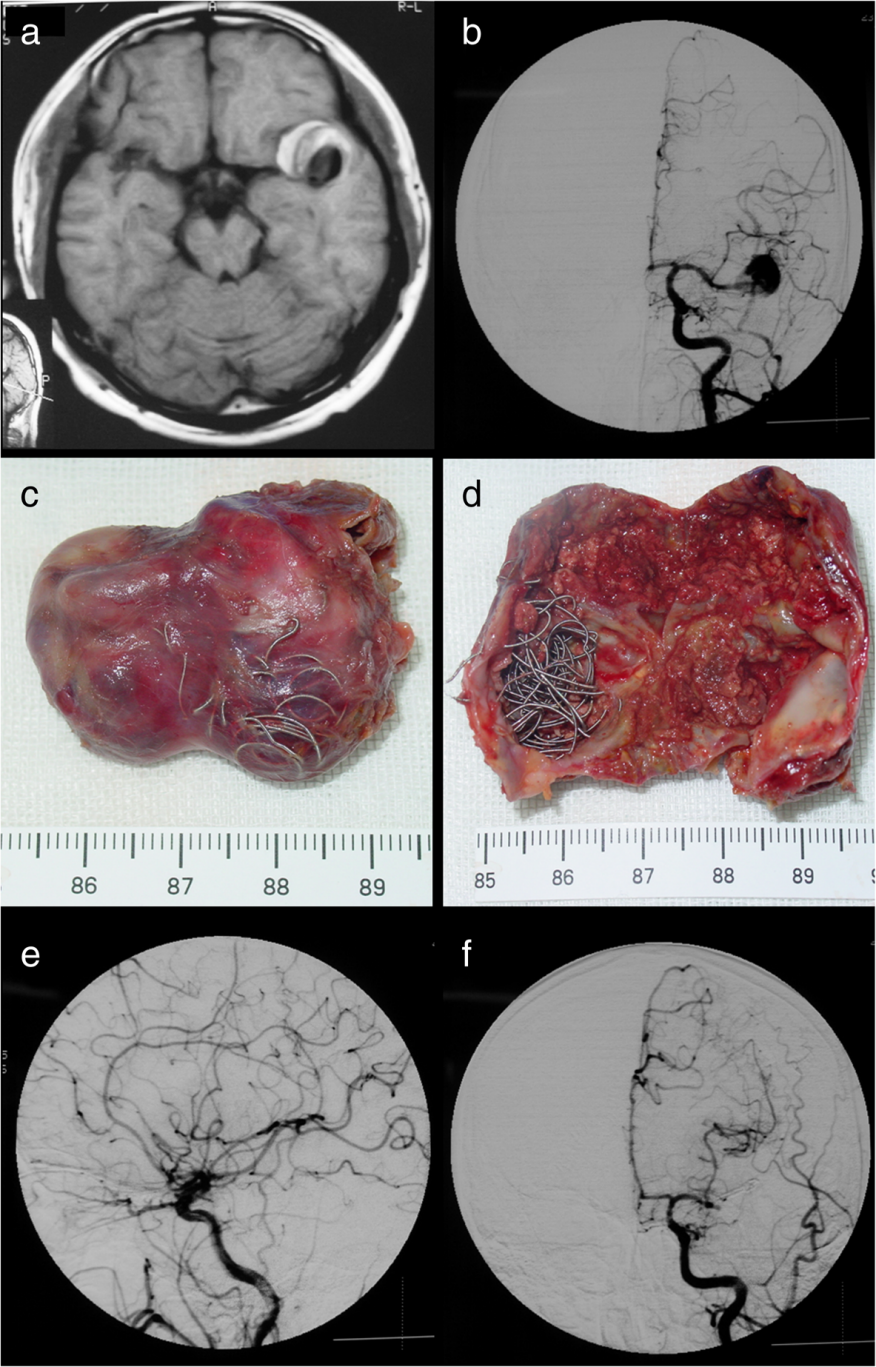
Patient 7. A 33-year-old man presented with a recurrent aneurysm 15 months after initial coiling (**a**, **b**). The coiled mass was completely removed (**c**, **d**). Coil extrusion through the aneurysm wall (**c**) and coil compaction (**d**) can be seen. Postoperative follow-up DSA showed complete obliteration of the recurrent aneurysm (**e**, **f**)

**Fig. 7:**
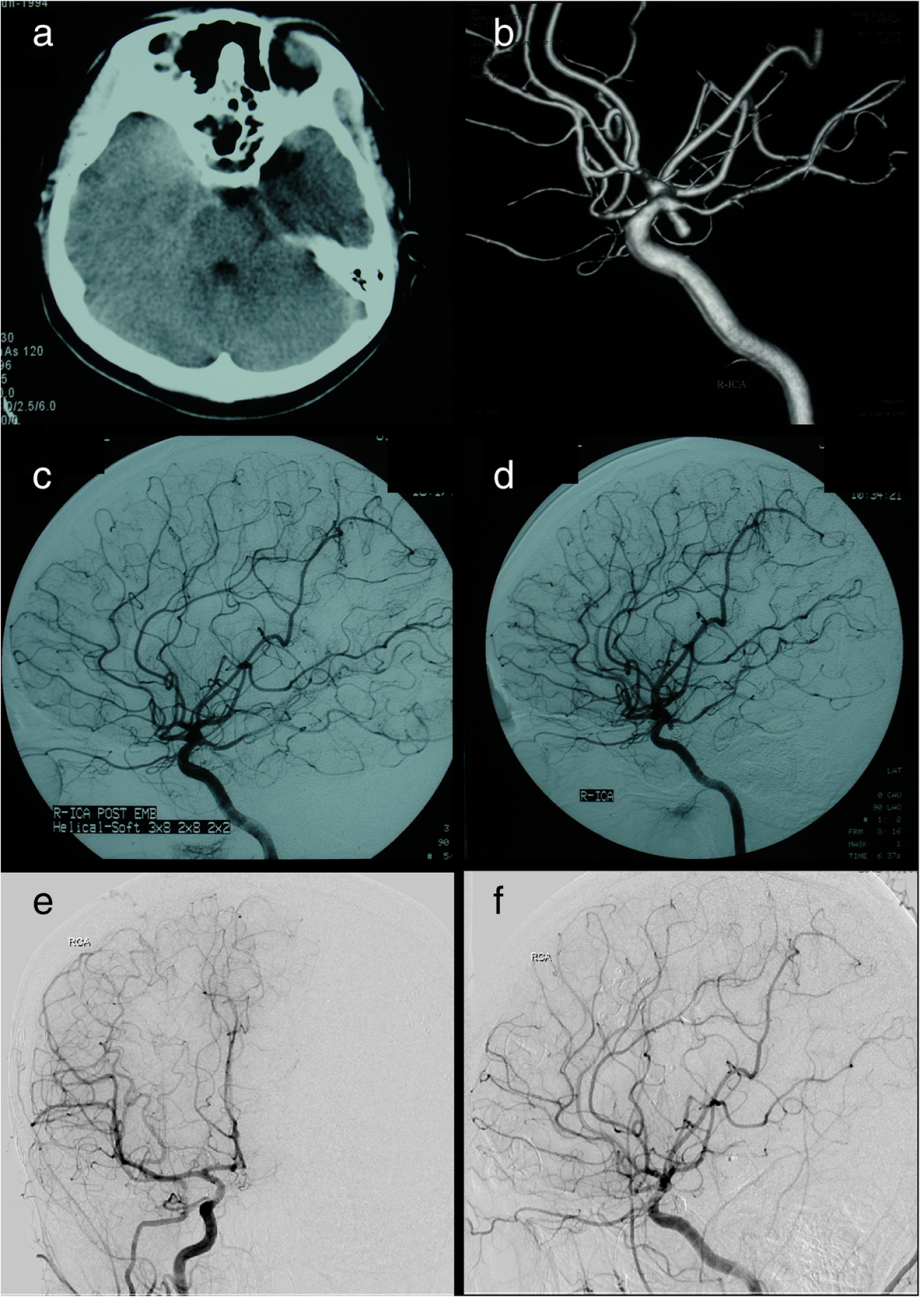
Patient 28. A 16-year-old woman presented with a ruptured right posterior communicating artery aneurysm (**a**, **b**). The aneurysm was completely obliterated with initial coiling (**c**). The follow-up DSA showed aneurysm recurrence 12 months after initial coiling (**d**). Microsurgical clipping without coil removal and postoperative DSA showed complete obliteration of the recurrent aneurysm (**e**, **f**)

**Fig. 8:**
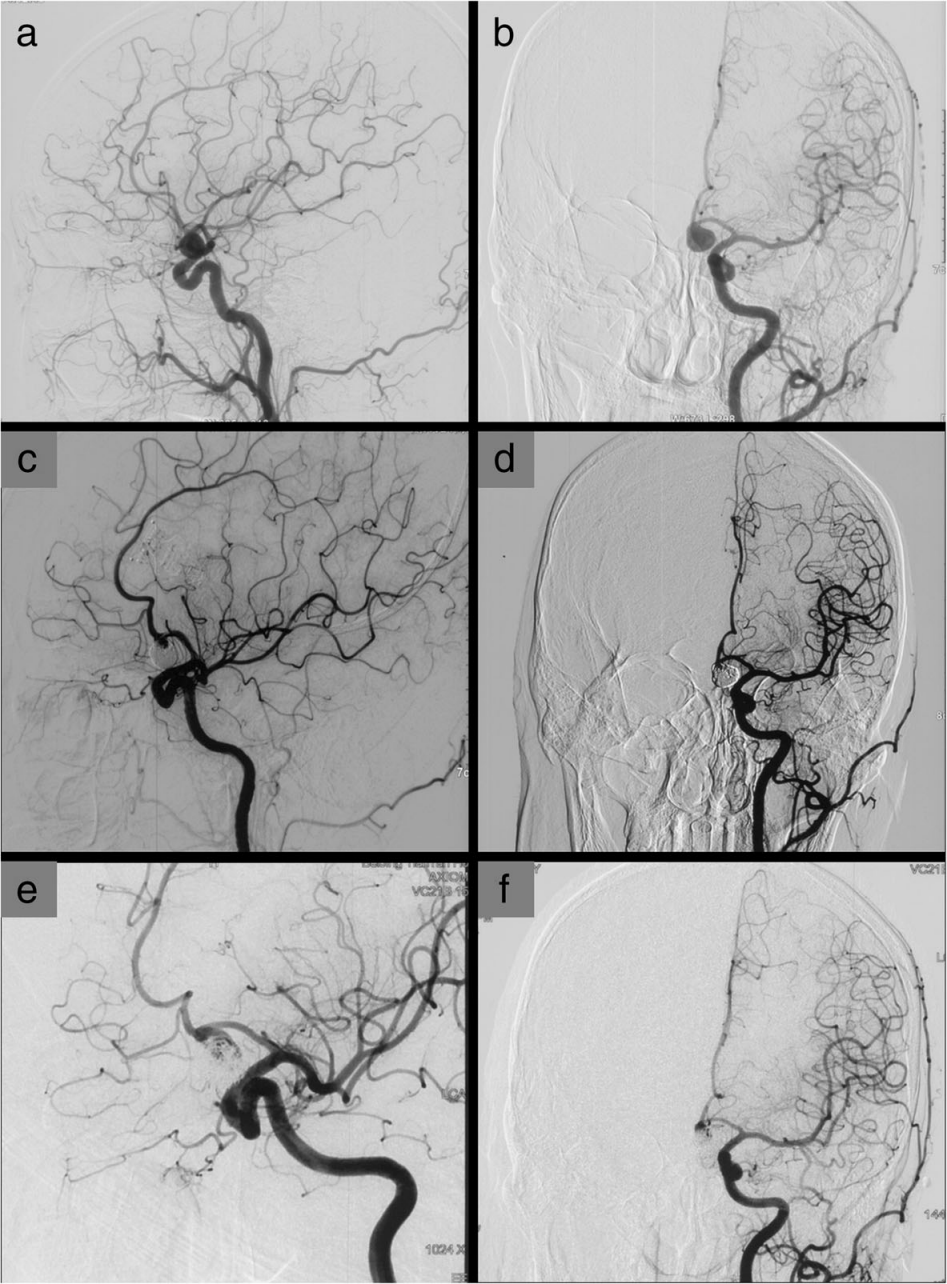
Patient 47. A 62-year-old woman presented with an unruptured AcomA aneurysm (**a**, **b**). Complete obliteration was achieved after initial coiling (**c**, **d**). The patient experienced visual failure 9 months after initial coiling and DSA showed an aneurysm recurrence (**e**, **f**)

**Fig. 9:**
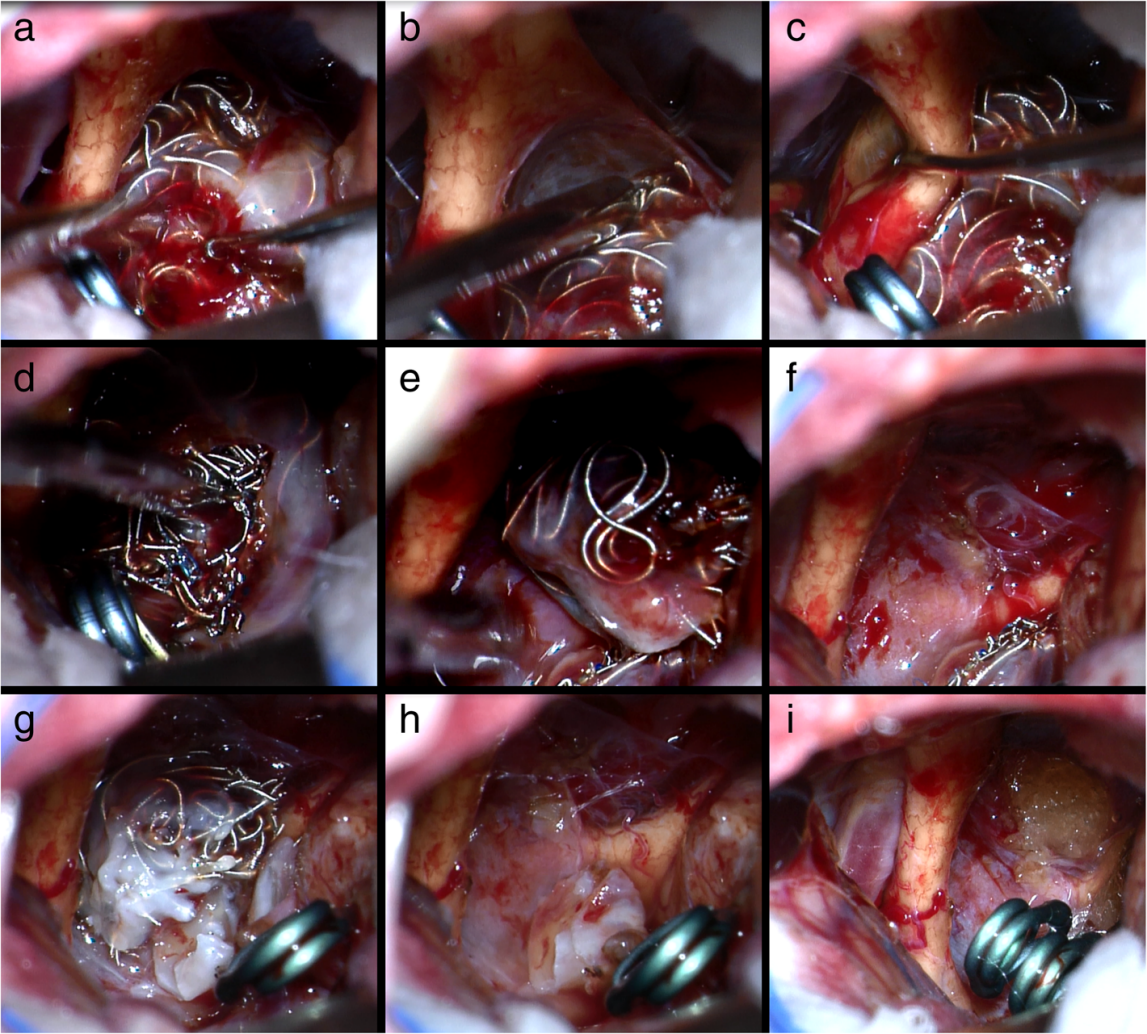
Intraoperative results of patient 47. Coil extrusion can be observed during surgery (**a**–**c**). The optic chiasm was severely compressed by the coiled mass (**a**–**c**). After exposure and control of the proximal and distal vessels of the aneurysm, careful dissection was performed to free the coiled mass from surrounding structures. The coiled mass was transected (**d**, **e**) and half of the coiled mass was removed. (**f**) The residual half of coiled mass become more mobile and there was adequate neck for clipping. After clipping with one clip, the residual half of coiled mass was removed (**g**, **h**). Another clip was placed next to the first clip (**i**)

Ultimately, during the 17-year period, 48 patients with 48 aneurysms underwent microsurgical management of incompletely obliterated or recurrent aneurysms previously treated by coiling. The mean follow-up period was 78.9 months (range, 10 months to 190 months). The medical records, radiographic studies, operative results, and follow-up data were reviewed retrospectively (Tables 1 and 2). We presented in this article six illustrations (patient 1, 2, 5, 7, 28 and 47; Figs. 1, 2, 3, 4, 5, 6, 7, 8, and 9).

**Table 1:** Preoperative data of patient characteristics

Patient no.	Age (yr)/sex	Presentation	HH	Aneurysm location	Side	Size (mm)	Coiling result	Recurrence	Latency
1	47/F	SAH	2	MCA	R	25	Complete occlusion	Angiographic	36
2	12/M	SAH	2	A1 ACA	L	15	Complete occlusion	Angiographic	3
3	38/M	SAH	2	PComA	R	12	Incomplete occlusion	–	–
4	64/F	SAH	3	PComA	L	17	Complete occlusion	SAH	18
5	3/M	Hemiplegia	0	MCA	L	26	Complete occlusion	Angiographic	11
6	64/M	SAH	2	PComA	R	12	Slight neck remnant	Mass effect	21
7	33/M	SAH	2	MCA	L	50	Complete occlusion	Mass effect	15
8	16/M	SAH	3	P2 PCA	L	40	Complete occlusion	Mass effect	28
9	42/F	SAH	2	PComA	R	10	Complete occlusion	Angiographic	10
10	45/F	Headache	0	ICA-OA	L	20	Complete occlusion	Angiographic	30
11	46/F	SAH	2	MCA	R	7	Slight neck remnant	Angiographic	18
12	63/F	SAH	3	PComA	L	6	Complete occlusion	Angiographic	38
13	49/M	SAH	2	AComA	M	30	Incomplete occlusion	–	–
14	46/M	SAH	2	AComA	M	25	Complete occlusion	Mass effect	13
15	34/F	Trauma	0	PComA	L	6	Slight neck remnant	Angiographic	15
16	55/F	SAH	2	PComA	L	10	Complete occlusion	Angiographic	16
17	38/M	SAH	1	PComA	R	28	Complete occlusion	Angiographic	20
18	28/F	SAH	1	A2 ACA	R	6	Slight neck remnant	Angiographic	12
19	68/F	SAH	2	PComA	R	8	Complete occlusion	Angiographic	5
20	60/M	SAH	1	A3 ACA	R	11	Complete occlusion	SAH	30
21	47/F	Headache	0	ICA-OA	L	20	Complete occlusion	Mass effect	12
22	53/M	SAH	1	AComA	M	5	Slight neck remnant	Angiographic	16
23	68/F	SAH	4	MCA	R	8	Complete occlusion	SAH	10
24	42/M	SAH	4	PComA	L	25	Complete occlusion	Angiographic	7
25	47/F	SAH	3	AComA	M	16	Complete occlusion	Angiographic	11
26	42/M	SAH	3	AComA	M	12	Incomplete occlusion	–	–
27	20/M	Trauma	0	PComA	L	15	Complete occlusion	Mass effect	28
28	16/F	SAH	1	PComA	R	8	Complete occlusion	Angiographic	12
29	51/M	SAH	3	PComA	R	5	Slight neck remnant	Angiographic	53
30	56/F	SAH	3	AComA	M	12	Complete occlusion	Mass effect	36
31	57/F	SAH	2	MCA	L	10	Incomplete occlusion	–	–
32	43/M	SAH	2	AComA	M	8	Complete occlusion	Angiographic	36
33	54/M	SAH	2	AComA	M	12	Complete occlusion	Mass effect	18
34	52/M	SAH	1	MCA	R	18	Incomplete occlusion	–	–
35	48/M	SAH	1	A3 ACA	R	9	Complete occlusion	SAH	4
36	49/M	SAH	1	AComA	M	5	Incomplete occlusion	–	–
37	37/M	SAH	2	MCA	R	8	Complete occlusion	Angiographic	60
38	45/M	SAH	2	P2 PCA	R	10	Slight neck remnant	Angiographic	12
39	47/F	SAH	2	PComA	R	6	Complete occlusion	Angiographic	36
40	55/F	SAH	1	PComA	R	7	Slight neck remnant	Angiographic	6
41	45/M	SAH	2	AComA	M	14	Complete occlusion	Angiographic	9
42	55/M	SAH	1	A2 ACA	R	10	Complete occlusion	Angiographic	12
43	63/F	SAH	3	PComA	R	13	Complete occlusion	Angiographic	20
44	51/M	SAH	3	AComA	M	8	Complete occlusion	Angiographic	12
45	59/F	SAH	1	PComA	L	17	Complete occlusion	Angiographic	75
46	61/F	SAH	3	PComA	R	12	Complete occlusion	Angiographic	12
47	62/F	Headache	0	AComA	M	15	Complete occlusion	Mass effect	9
48	57/M	SAH	2	A1 ACA	R	6	Complete occlusion	Angiographic	5

*yr* years, *F* female, *M* male, *SAH* subarachnoid hemorrhage, *H-H* Hunt and Hess grade, *MCA* middle cerebral artery, *ACA* anterior cerebral artery, *PComA* posterior communicating artery, *PCA* posterior cerebral artery, *ICA-OA* internal carotid artery–ophthalmic artery, *AComA* anterior communicating artery, *R* right, *L* left, *M* middle, *Latency* time interval from initial coiling to recurrence

**Table 2:** Microsurgical results and follow-up data

Patient no.	Age (yr)/sex	Aneurysm location	Coil compaction or extrusion	Microsurgery	Aneurysm occlusion	GOS		Outcome	Follow-up (m)
						Preop	Final		
1	47/F	MCA	Extrusion	Clip + TCR	Complete	5	5	Unchanged	190
2	12/M	A1 ACA	Compaction	Clip + TCR	Complete	5	5	Unchanged	182
3	38/M	PComA	None	Clip + TCR	Complete	5	5	Unchanged	168
4	64/F	PComA	Extrusion	Clip	Complete	3	3	Unchanged	159
5	3/M	MCA	Compaction	Clip + TCR	Complete	4	5	Improved	150
6	64/M	PComA	Extrusion	Clip + PCR	Complete	4	5	Improved	146
7	33/M	MCA	Extrusion	Clip + TCR	Complete	4	4	Unchanged	139
8	16/M	P2 PCA	Extrusion	Clip + TCR	Complete	3	3	Unchanged	133
9	42/F	PComA	Extrusion	Clip	Complete	4	4	Unchanged	128
10	45/F	ICA-OA	Compaction	Clip + TCR	Complete	3	3	Unchanged	125
11	46/F	MCA	Extrusion	Clip	Complete	5	5	Unchanged	120
12	63/F	PComA	Extrusion	Clip	Complete	3	4	Improved	116
13	49/M	AComA	None	Clip + TCR	Complete	5	5	Unchanged	110
14	46/M	AComA	Extrusion	Clip + PCR	Complete	4	4	Unchanged	107
15	34/F	PComA	Compaction	Clip	Complete	5	5	Unchanged	105
16	55/F	PComA	Extrusion	Clip	Complete	5	5	Unchanged	99
17	38/M	PComA	Extrusion	Clip + PCR	Complete	5	5	Unchanged	95
18	28/F	A2 ACA	Extrusion	Clip	Complete	5	4	Worse	92
19	68/F	PComA	Extrusion	Clip + PCR	Complete	5	5	Unchanged	90
20	60/M	A3 ACA	Compaction	Clip + PCR	Complete	3	3	Unchanged	84
21	47/F	ICA-OA	Compaction	Clip + TCR	Complete	5	5	Unchanged	80
22	53/M	AComA	Extrusion	Clip	Complete	5	5	Unchanged	78
23	68/F	MCA	Extrusion	Clip + PCR	Complete	3	2	Worse	75
24	42/M	PComA	Extrusion	Clip + PCR	Complete	4	3	Worse	70
25	47/F	AComA	Compaction	Clip + TCR	Complete	5	5	Unchanged	68
26	42/M	AComA	None	Clip + TCR	Complete	5	5	Unchanged	67
27	20/M	PComA	Compaction	Clip + TCR	Complete	5	5	Unchanged	63
28	16/F	PComA	Extrusion	Clip	Complete	4	5	Improved	60
29	51/M	PComA	Extrusion	Clip	Complete	5	5	Unchanged	60
30	56/F	AComA	Extrusion	Clip + TCR	Complete	5	5	Unchanged	59
31	57/F	MCA	None	Clip + TCR	Complete	5	5	Unchanged	55
32	43/M	AComA	Extrusion	Clip	Complete	4	4	Unchanged	53
33	54/M	AComA	Extrusion	Clip + PCR	Complete	4	4	Unchanged	50
34	52/M	MCA	None	Clip + TCR	Complete	4	4	Unchanged	48
35	48/M	A3 ACA	Extrusion	Clip + PCR	Complete	3	4	Improved	47
36	49/M	AComA	None	Clip	Complete	5	4	Worse	43
37	37/M	MCA	Extrusion	Clip + PCR	Complete	5	5	Unchanged	39
38	45/M	P2 PCA	Extrusion	Clip + PCR	Complete	5	5	Unchanged	36
39	47/F	PComA	Extrusion	Clip	Complete	4	4	Unchanged	32
40	55/F	PComA	Extrusion	Clip	Complete	5	5	Unchanged	29
41	45/M	AComA	Compaction	Clip + PCR	Complete	5	5	Unchanged	28
42	55/M	A2 ACA	Compaction	Clip + TCR	Complete	5	5	Unchanged	23
43	63/F	PComA	Extrusion	Clip + PCR	Complete	5	4	Worse	21
44	51/M	AComA	Compaction	Clip	Complete	5	5	Unchanged	17
45	59/F	PComA	Extrusion	Clip + PCR	Complete	5	5	Unchanged	14
46	61/F	PComA	Extrusion	Clip + TCR	Complete	5	5	Unchanged	12
47	62/F	AComA	Extrusion	Clip + TCR	Complete	4	5	Improved	12
48	57/M	A1 ACA	Compaction	Clip	Complete	5	5	Unchanged	10

*yr* years, *F* female, *M* male, *SAH* subarachnoid hemorrhage, *MCA* middle cerebral artery, *ACA* anterior cerebral artery, *PComA* posterior communicating artery, *PCA* posterior cerebral artery, *ICA-OA* internal carotid artery–ophthalmic artery, *AComA* anterior communicating artery, *TCR* total coil removal, *PCR* partial coil removal, *GOS* Glasgow Outcome Scale, *Preop* preoperative, *Final* at the final follow-up

As illustrated in Table 1, this series included 22 female and 26 male patients with a mean age of 46.5 years (range, 3 to 68 years) at the time of surgery. Forty-two patients (87.5%) had a history of aneurysmal subarachnoid hemorrhage (SAH), with Hunt and Hess grade I in 11 patients, grade II in 19 patients, grade III in 10 patients, and grade IV in 2 patients. The aneurysms in the other 6 patients were incidentally found after head trauma or chronic headaches. Eight aneurysms were classified as giant (at least 25 mm), 23 were classified as large (10–24 mm), and 17 were classified as small (less than 10 mm). Forty-six aneurysms were located in the anterior circulation and 2 were located in the posterior circulation.

### Endovascular management

The initial endovascular coiling was performed without adjuvant techniques (balloon-assisted or stent-assisted coiling). After initial coiling, complete obliteration was achieved in 34 patients, slight neck remnant (90–99% occlusion) in 8 patients, and incomplete obliteration (< 90% occlusion) in 6 patients (Table 1). Routine follow-up cerebral angiography was performed for those 42 patients with completely or nearly completely obliterated aneurysms after initial embolization. All the 6 patients with initially incompletely obliterated aneurysms were referred for microsurgical management within 1 month after initial endovascular coiling and no patients experienced rebleeding during this time interval. Four patients presented with rebleeding at the time of 4 months, 10 months, 18 months, and 30 months respectively after initial coiling (Table 1). There were 29 patients without symptomatic deterioration after coiling showed significant angiographic recurrence on follow-up angiography. Nine patients experienced symptomatic mass effects, including 4 AComA aneurysms causing visual deterioration, 2 PComA aneurysms causing nerve III palsy, 1 OphA aneurysm with visual deterioration, 1 PCA aneurysm with mild hemiplegia, and 1 MCA aneurysm with secondary epilepsy. Cerebral angiography of these aneurysms of with mass effects or rebleeding also showed significant aneurysm recurrence. The mean time from initial coiling to recurrence (recurrent latency) for the 42 patients was 20.2 months (range, 3 months to 75 months, Table 1). The hemorrhage rate after aneurysm coiling for our series was 8.3% (4 of 48 patients).

### Microsurgical management

One patient (patient 5) underwent microsurgical clipping 4 months after recoiling. Other patients underwent microsurgical clipping within 2 months after the diagnosis of incompletely coiled or recurrent aneurysms. During surgery, most of the coiled aneurysms had a thin, translucent, and fragile wall (Figs. 3, 5, and 9). Of the 6 patients with initially incompletely coiled aneurysms, due to short time interval from coiling to surgery, no one was observed with coil compaction or coil extrusion through the aneurysm wall into the subarachnoid space (Table 2). Of the 42 recurrent coiled aneurysms, 71% (30/42) were observed with coils extrusion into the subarachnoid space (Figs. 6 and 9) and 29% (12/42) had coil compaction (Table 2, Figs. 3 and 5). Partial or total removal of coils was considered to facilitate microsurgical clipping when the coiled aneurysms caused mass effects, the coils protruded into the aneurysm neck, or no adequate neck remnant for clipping. As listed in Table 2, of all the 48 patients, direct surgical clipping without coils removal was accomplished in 16 patients (33.3%; Fig. 7). Surgical clipping with partial coil removal was achieved in 14 patients (29.2%). Surgical clipping with total removal of coils was performed in 18 patients (37.5%; Figs. 1, 3, 5, 6, and 7). Bypass surgery, trapping, or wrapping was not performed in any of our patients.

### Follow-up

Postoperative angiography revealed that all the previously coiled aneurysms in our series were completely obliterated after microsurgical clipping. No patient died during the postoperative follow-up period. Two patient experienced transient neurological deficit that resolved completely at 3 and 5 months respectively after surgery. Postoperative hydrocephalus occurred in 3 patients, of which 2 patients received V-P shunt and the other one was relatively stable by observation. At the final follow-up, good outcomes (GOS of 4 or 5) were achieved in 87.5% (42/48) of the patients. Five patients had a GOS of 3 and one had a GOS of 2. Compared with the preoperative GOS scores, the patient neurological outcome (GOS) at the final evaluation was improved or unchanged in 89.6% and worse in 10.4%.

## Discussion

Endovascular treatment of intracranial aneurysms has developed rapidly since its first introduction in 1974 [30]. Endovascular coiling has become a routine technique for intracranial aneurysms since the development of Guglielmi detachable coils in the 1990s [13]. With the increasing number of patients undergoing aneurysm endovascular coiling, the population of patients with incompletely coiled or recurrent aneurysms continues to increase. Aneurysm recurrence after initial endovascular therapy has been documented to occur at a rate of 3.6 to 40% [5, 7, 17, 23, 25]. However, there are no established guidelines regarding the treatment modality for these previously coiled aneurysms. Treatment options include repeat endovascular treatment, microsurgical clipping, bypass surgery, and parent artery occlusion. In the past 17 years, a series of 48 consecutive patients with incomplete coiled or recurrent aneurysms were treated with microsurgical clipping. We presented our experience in treating this setting of aneurysms.

### Microsurgical technique

Compared with those uncoiled aneurysms, previously coiled aneurysms cannot be softened, collapsed, or deflated easily [20, 34]. Coiled aneurysms may become thrombotic and hardened, which may add difficulties to surgical procedure during the process of dissecting the aneurysm from surrounding structures. Coil migration into the aneurysm neck and parental or collateral arteries makes surgical clipping more difficult. Coil extrusion into the subarachnoid space is also a challenging issue during dissection.

For the 6 patients with incompletely coiled aneurysms after initial embolization, the coiled aneurysms were easily dissected free from surrounding structures. All the 6 incompletely coiled aneurysms were clipped within 1 month after initial coiling when coils remain mobile, the thrombus is soft, and the coil mass was not compacted. We recommend early microsurgical intervention for incompletely coiled aneurysms to prevent aneurysm rupture. In our series, for the five incompletely coiled aneurysms with the size > 10 mm, total coil removal was performed to prevent potential mass effects at a later stage after surgery. We do not recommend coil removal for those coiled aneurysms with the size < 10 mm unless the coils protrude into the neck or there is no adequate neck remnant for clipping.

In contrast to incompletely coiled aneurysms, recurrent aneurysms were often hard and less mobile. The thrombus sometimes makes it difficult to mobilize and clip these recurrent aneurysms. Coil compaction, migration, or extrusion adds difficulty for dissecting and clipping process. To achieve successful microsurgical clipping, we recommend obtaining early proximal control of the aneurysms.

### Coil extraction or not

For the patients with coils migration into the aneurysm neck or the parental artery, it is necessary to remove the coils to get enough space to clip the aneurysm neck. For patients with coiled aneurysms that caused mass effects, partial or total coil removal should be performed. Partial or total coil removal can provide adequate decompression of the aneurysm sac, relieve the mass effect caused by coiled mass, and help to achieve successful neck clipping.

Removal of coils from coiled aneurysms is technically challenging. Controversies exist regarding the danger and necessity of coil extraction during surgery [6, 12, 22, 26, 28, 29, 34]. In our series, microsurgical clipping with partial or complete coil removal was performed in 67% (32/48) of the patients. In the literature review of 375 incompletely coiled or recurrent aneurysms, only 13% of coils were extracted and most underwent direct microsurgical clipping without coil removal [2]. Some authors recommend systematic extraction of coils to achieve successful clipping, even if the coils have protruded into the aneurysm neck or parent arteries [22, 28, 31]. While other authors emphasize the danger of removing coils from previously coiled aneurysms [10, 12, 29]. Horowitz et al. initially planned to perform coil extraction as a goal in all cases, but only achieved in 40% of reported cases. As a result, they favored direct clipping whenever possible to avoid damage to the parent artery [2, 15]. Thornton et al. performed coil extraction in 81% in their series [31]. In fact, most (64%) of their cases underwent microsurgical management within 2 weeks after endovascular coiling. Coil extraction is relatively easier if the coils are placed “recently” [31]. In our series, all the six patients with incompletely coiled aneurysms underwent surgical clipping within 1 month after coiling and total coil extraction was achieved in five patients (83.3%). Dorfer reported a patient with recurrent aneurysm 4 years after embolization. The coil removal was attempted and resulted in aneurysm rupture at the neck, leading to sacrificing the ICA and permanent morbidity [12]. In our series, we tried to remove the coiled mass when it prevents satisfactory clipping of the aneurysm. In such situations as coil-caused immobilization, no adequate neck for clipping, coil protrusion into the neck or parental arteries, coil-induced mass effects and potential mass effects, the coiled mass inside the aneurysm has to be removed to appropriately apply clips and achieve favorable outcomes. According to the literature and our experience, if coil removal is deemed necessary but cannot be done safely, partial coil removal is recommended to facilitate clipping [2]. As a result, partial coil removal to achieve proper clip placement or decompress mass effect was done in 29.2% (14/48) of our cases. Alternatively, to facilitate surgical clipping, some authors recommend simple opening of the aneurysm dome to extrude the coils partially (without coil removal) [1, 2, 11, 20, 33, 35, 36]. As to the literature and our experience, if coil removal is attempted, it is essential to achieve early control of the proximal afferent and distal efferent vessels. Vigorous traction should be avoided during dissection to reduce the risk of aneurysm sac separation from the parental artery [24, 31, 32].

Aneurysm location is also a factor that influences coil removal and surgical clipping. Regarding our experience, most of the incompletely coiled and recurrent aneurysms were located in the anterior circulation and only two were located in the P2 segment of PCA. The locations such as ACA, AcomA MCA, and PcomA provided easier access and enough space for aneurysm dissection, coil removal, and surgical clipping in most cases. For the two patients with coiled aneurysms in the P2 segment of the PCA, the coiled mass was totally removed in one patient and partially removed in the other patient. None of the coiled aneurysms in our series were located in such confining anatomic locations as the basilar trunk, basilar apex, or carotid bifurcation [3, 4, 19]. For coiled aneurysms in such locations, surgical clipping is extremely challenging. Recoiling may be the preferred choice.

Aneurysm size is also a factor that influences the coil removal and surgical clipping in our series. In our experience, for small-sized (< 10 mm) coiled aneurysms, if they have adequate neck for clipping, without coils in the neck or mass effects, we recommend direct surgical clipping without coil removal. If they have coils in the neck, or mass effects, or no adequate neck remnant for clipping, partial or total coil removal should be performed to achieve successful surgical clipping. For those coiled aneurysms with the size > 10 mm, we recommend partial or total coil removal to facilitate surgical clipping, relieve mass effects, or prevent potential postsurgical mass effects. However, all the clipping procedures should not be performed at the expense of patients’ safety.

In our series, for aneurysms with coil protrusion into the neck or no adequate neck for clipping, we had to remove partial or total coiled mass to achieve complete occlusion of the aneurysms or prevent parent artery stenosis. Izumo reported a combination using of a fenestrated clip and a second type of clip in four of six patients to successfully occlude the recurrent aneurysms with coils in the neck, and no patients needed coil removal [16]. This technique is reasonable in treating recurrent aneurysms. However, in the practice of treating coiled or recurrent aneurysms, there are times when coil removal is unavoidable. Considering the difficulty of coil removal and the potential complications of surgical clipping created by the presence of coils in the aneurysm neck, some authors recommend observation for these aneurysms with coils in the neck until additional coil compaction facilitates clipping [2, 11, 14, 20, 31]. We agree with their recommendations. However, these patients should be followed clinically and angiographically with close observation.

### Surgical outcomes

In the literature review, the mean obliteration rate after microsurgical clipping of incompletely coiled or recurrent aneurysms was 93% and 42% of the authors reported 100% obliteration [2]. A mean postsurgical GOS of 4.4 was achieved and most cases experienced an unchanged postsurgical GOS score compared with their preoperative state [2]. In our series, the obliteration rate was 100%. The mean GOS at the final follow-up was 4.5, and 90% had an unchanged or improved GOS after surgical clipping. The outcome data in the literature and our study showed that microsurgical clipping of incompletely coiled or recurrent aneurysms can achieve high obliteration rate and favorable clinical outcomes with low morbidity and mortality in selected patients.

### Limitations

We retrospectively reviewed our 17-year experience of treating previously coiled or recurrent aneurysms. Our series represent a highly selected group of patients who sought for microsurgical clipping of their coiled or recurrent aneurysms after initial endovascular coiling. Although successful microsurgical clipping was achieved in all the patients, the aneurysms in our series were relatively easily accessible. We must admit that more complex aneurysms with high surgical difficulties should be managed with recoiling, bypass surgery, trapping, or wrapping.

## Conclusions

Microsurgical clipping is effective for incompletely coiled or recurrent aneurysms after initial coiling. For recurrent aneurysms with coils in the neck, inadequate neck for clipping or mass effects on surrounding structures, partial coil removal, or total removal of the coil mass can facilitate surgical clipping and successful obliteration of the aneurysms. However, coil extraction is challenging and should be decided and performed cautiously by experienced vascular neurosurgeons.

## Abbreviations

CARAT: Cerebral aneurysm rerupture after treatment
DSA: Digital subtraction angiography
ISAT: International subarachnoid aneurysm trial
MRA: Magnetic resonance angiography
